# Supplementary figures and images for: Effects of N-glycan modifications on spike expression, virus infectivity, and neutralization sensitivity in ancestral compared to Omicron SARS-CoV-2 variants
Source: PLoS Pathog. 2023 Nov 9;19(11):e1011788. doi: 10.1371/journal.ppat.1011788 (PMC10662749; doi:10.1371/journal.ppat.1011788)

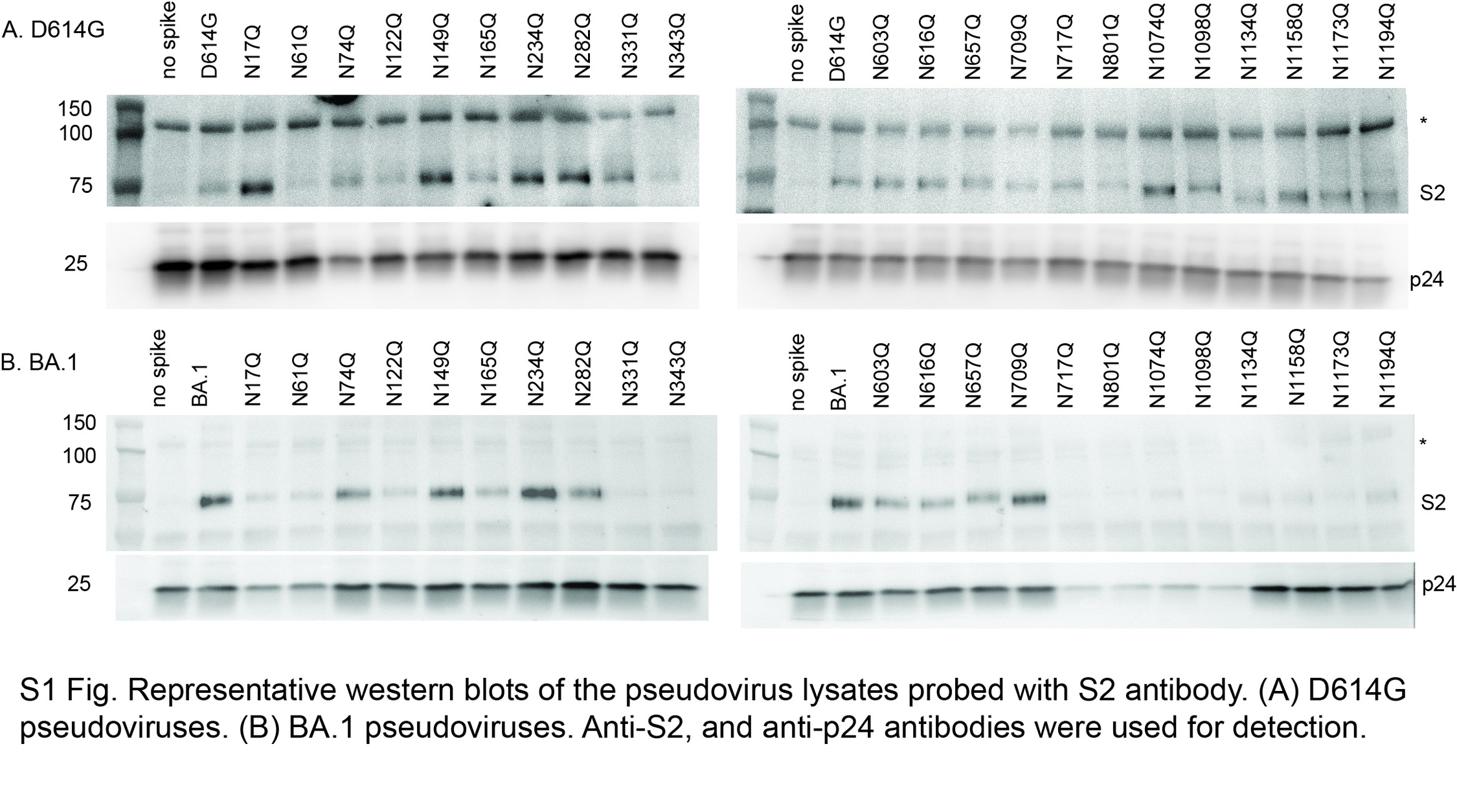

Supplement: S1 Fig — (A) D614G pseudoviruses. (B) BA.1 pseudoviruses. Anti-S2, and anti-p24 antibodies were used for detection. (TIF) [file ppat.1011788.s001.tif]

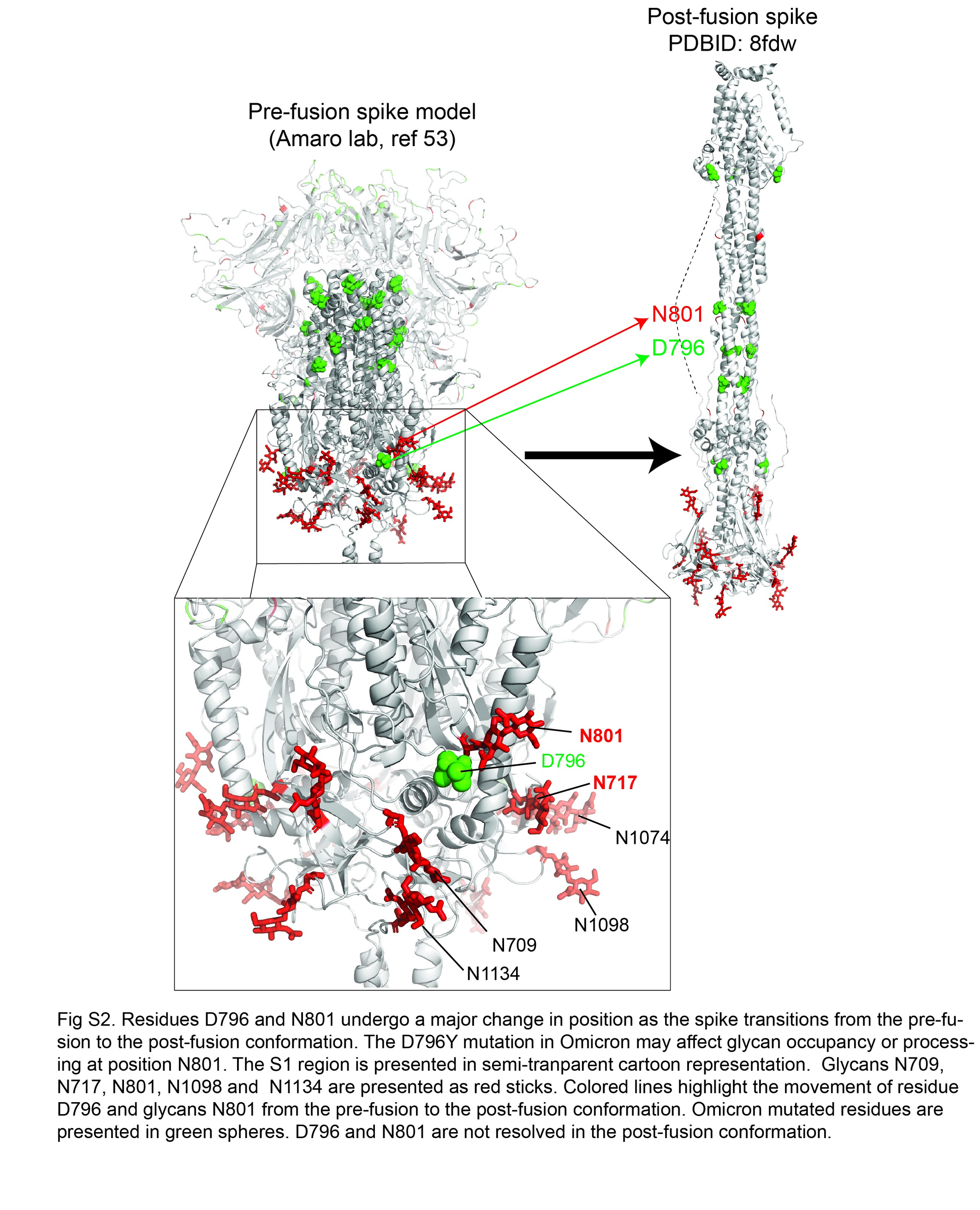

Supplement: S2 Fig — The D796Y mutation in Omicron may affect glycan occupancy or processing at position N801. The S1 region is presented in semi-transparent cartoon representation. Glycans N709, N717, N801, N1098 and N1134 are presented as red sticks. Colored lines highlight the movement of residue D796 and glycans N801 from the pre-fusion to the post-fusion conformation. Omicron mutated residues are presented in green spheres. D796 and N801 are not resolved in the post-fusion conformation. (TIF) [file ppat.1011788.s002.tif]

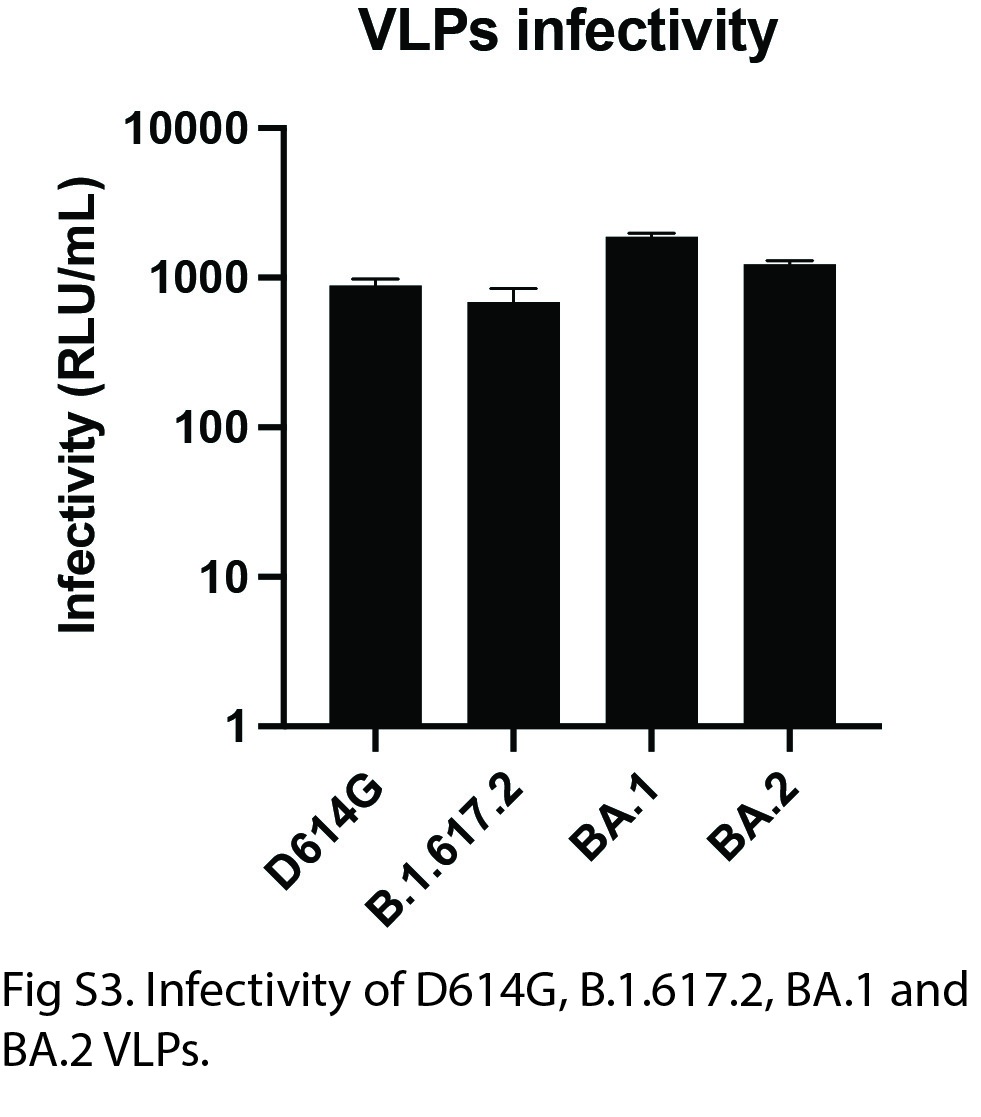

Supplement: S3 Fig — (TIF) [file ppat.1011788.s003.tif]
