# Supplementary material for: Effects of N-glycan modifications on spike expression, virus infectivity, and neutralization sensitivity in ancestral compared to Omicron SARS-CoV-2 variants
Source: PLoS Pathog. 2023 Nov 9;19(11):e1011788. doi: 10.1371/journal.ppat.1011788 (PMC10662749; doi:10.1371/journal.ppat.1011788)
Supplement: Attachment — Submitted filename: PPATHOGENS-D-23-00772 Bhiman Review.docx [file ppat.1011788.s005.docx]

**PPATHOGENS-D-23-00772**

**D614G and Omicron SARS-CoV-2 variant spike proteins differ in the effects of N-glycan modifications on spike expression, virus infectivity, and neutralization by some therapeutic antibodies**

**Overall assessment and summary**

This manuscript represented a substantial body of work aimed at describing the impact of glycosylation on the spike protein of SARS-CoV-2 on viral infectivity, spike processing and neutralization sensitivity. The authors describe how despite high conservation of glycosylation sites between pre- and post-Omicron variants, modification of these glycan impact these variants uniquely. Removal of glycosylation sites in the S2, namely at N717 and N801, have profound impacts on Omicron variant pseudovirus infectivity, while only removal of N61 in S1 reduces infectivity of the D614G variant. The authors show that the presence of high mannose glycans ablates infectivity of Omicron variants, while having limited impact on pre-Omicron variants. Lastly the authors show that class 3 RBD mAbs are impacted by glycan modifications that favour the RBD up conformation of spike, while class 1 RBD mAbs are impacted by those modifications that favour the RBD down conformation.

What is lacking for me, is that the authors have not linked the findings from each set experiments to present a model or mechanism or reason for why, as their data suggests, there are functionally-dependent differences in glycosylation requirements for pre versus post-Omicron variants. The conclusion paragraphs that they authors have presented largely represents a summary of the results with implications that are very technical in their application.

**Recommendation**

There are key details regarding the methodology that have been excluded from the current manuscript, making it difficult to evaluate the presented results. Therefore, I would recommend that these are included and the manuscript undergoes a second round of review.

**Major comments**

**Methods**

Line 508

Given that the infectivity and relative infectivity measures are so critical to results, please provide more details as to how infectivity was measured and how relative infectivity was calculated. Is infectivity measured as the RLUs produced by a specific virus transfection? Were different aliquots or the same aliquot used for the p24 western blot and virus infectivity measures? Were multiple lots grown on different days compared?

VLP generation

Please include a section detailing how the SARS-CoV-2 VLPs were generated and quality checked.

**Results**

Line 436-444

This section comparing lentivirus to VLP is difficult to assess given the lack of details as to how the VLPs were generated. Regardless, results for a control antibody, that is able to neutralise the VLPs at greater than 30% maximum inhibition is required to demonstrate robustness of the assay.

**Discussion**

The authors have included multiple points in their results section that could form the basis of a well-constructed discussion, which is currently lacking and which I believe will force the authors to tie together the many different points into some mechanistic and biologically relevant implications, rather than simply a set of technical recommendations.

**Minor comments**

**Title**

I would recommend a modification to the title to read as “ Effects of N-glycan modifications on spike expression, virus infectivity and neutralization sensitivity in ancestral compared to Omicron SARS-CoV-2 variants”

Throughout the manuscript – “authentic virus”.

I would recommend that the authors refer to “authentic virus” as “live virus” or because in my opinion, there is nothing authentic about culturing SARS-CoV-2 in Vero E6 cells.

Line 52

Suggest to alter the sentence to read as follows “Changes in the overall glycan composition also reduced the infectivity of Omicron compared to D614G in the live or pseudovirus format.”

Line 67

The S2 subunit contains the transmembrane domain and contains other regions that do not span the transmembrane region. Therefore please modify the sentence to read “….and a non-covalently associated *viral fusion* subunit (S2).”

Line 119

Please clarify whether fold drop in infectivity was based on comparison to wild-type D614G (Fig 1C) or wild-type BA.1 (Figure 1D)?

Line 204

The authors state that D614G and BA.1 had similar levels of infectivity, however in the western blots in both Figure 2 and Figure 3, the S1 bands for BA.1 appear to be much darker than for D614G. Can the authors comment on this relative to the infectivity read out?

Line 227

The authors state that BA.1/BA/2 spikes depend on more “proper” glycosylation. Can the authors clarify what they mean by proper glycosylation or replace the term with “more processed glycans”?

**Figures**

Figure 1 legend

Please state what the dashed lines in panels C, D and E indicate?

Might make it easier for the reader if you indicate where the 10-fold and 100-fold difference in infectivity is for Fig 1, C, D and E.

Figure 3A

Please include labels for the various glycoforms eg high mannose/oligomannose, hybrid or complex. Alternately, because for eg Man_5-9_GlcNAc_2_ is referred to in the text, these types of labels may also help orient the reader.

Figure 3 legend

There appears to be an error in the description for panel B and C. I assume panel B to represent results for multiple variants infectivity on the absence of glycosylation modifiers, however the current description appears to refer to panel C. In addition, the description for panel C only refers to mean and duplicates for each assay.
