# Supplementary material for: Effects of N-glycan modifications on spike expression, virus infectivity, and neutralization sensitivity in ancestral compared to Omicron SARS-CoV-2 variants
Source: PLoS Pathog. 2023 Nov 9;19(11):e1011788. doi: 10.1371/journal.ppat.1011788 (PMC10662749; doi:10.1371/journal.ppat.1011788)
Supplement: Attachment — Submitted filename: D-23-00772 response to reviewers.docx [file ppat.1011788.s006.docx]

**Response to Reviewers**

The reviewers appreciated the significant body of work presented in this manuscript, and felt that these data could add significantly to the field, and be of broad interest. However, they raise several textual and experimental concerns that should be addressed prior to potential acceptance. A major concern is the heavy reliance on data from pseudotyped viruses with very limited experiments performed in live virus assays. The methodology is sometimes light on details making the quality of the data difficult to fully assess. The reviewers also suggest that more effort and thought go into linking these site-specific data to a potential model or mechanism for these findings, rather than a conclusion which simply restates the data.

**Authors’ reply:** We appreciate that the reviewers thought the large body of work was significant and of broad interest. We also appreciate the additional comments and concerns and made revisions accordingly, which we believe improve the manuscript.

Reviewer's Responses to Questions

**Part I - Summary**
Please use this section to discuss strengths/weaknesses of study, novelty/significance, general execution and scholarship.

Reviewer #1: This study explores how glycan deletions/modifications affect Omicron BA.1 and D614G spike expression, processing, incorporation into pseudoviruses as well as virus infectivity and neutralization by antibodies. The main finding of this paper is the site-specific glycan deletions have a different effect on Omicron than for previous variants. Notably, the loss of glycosylation at residues 717 and 801 each conferred a loss of infectivity for Omicron but not for D614G or Delta variants, with the decreased infectivity correlated with decreased spike processing and incorporation into Omicron pseudoviruses. Further, the authors showed that complex glycans play a more important role in Omicron, with mannose-enriched Omicron pseudoviruses being non-infectious, whereas D614G or Delta pseudoviruses generated under similar conditions remained infectious. The authors also tested authentic SARS-CoV-2 grown with kifunensine which decreased titers more for BA.1.1 than for Delta or D614G. Some glycan deletions affect neutralization by antibodies at various regions differently in D614G vs. BA.1.

This study provide valuable information on spike biology about the role glycans play for different SARS-CoV-2 variants, particularly how their effect on the currently prevalent Omciron differs from previous variants. This is a technically sounds study and incorporates the higher throughput (and safer) pseudovirus neutralization assays with fewer but quality authentic coronavirus experiments with mutations of interest. The information about antibody binding with the glycan deletions will be useful for antibody development in the future.

**Authors’ reply:** We thank the reviewer highlighting the valuable information provided in our study.

Reviewer #2: A large number of studies have been performed in this paper, and they suggest that glycans at specific locations of Spike protein have a greater role in regulating omicron Spike function compared to D614G Spike. Omicron Spike is also more susceptible to inhibitors of glycosidases. Further, glycans may regulate viral susceptibility to neutralization. The findings are largely in line with previous published results, but they are valuable due to the differences noted related to the role of glycans in the original virus vs. the newer variants.

A main limitation of the study is that most of the work is focused on pseudotyped lentivirus which are processed differently compared to the actual virus. Thus, whether the functional modifications observed with lentivirus carry over to the authentic virus remains to be seen. This is particularly related to observations related to the incorporation of N717 and N801 Spike into the virion particles.

From a biomedical perspective, the most interesting result is related to the studies with kifunensine where the titer of the Omicron variant is dramatically reduced compared to D614G. For a paper to be published in PLOS Pathogens, I would recommend great effort to characterize this difference using additional assays related to CPE and plaque formation. A more careful investigation of this process can be of considerable basic science and translational value.

**Authors’ reply:** We thank the reviewer for noting the depth of our studies and the importance of the difference in glycan effects in Omicron compared to earlier variants. We appreciate the comment about additional assays for quantifying growth of live (authentic) viruses. As explained in more detail in the response in Part II, we used a focus-forming assay due to poor cytopathic effects of the Omicron variant in Vero E6 cells.

Reviewer #3: This manuscript represented a substantial body of work aimed at describing the impact of glycosylation on the spike protein of SARS-CoV-2 on viral infectivity, spike processing and neutralization sensitivity. The authors describe how despite high conservation of glycosylation sites between pre- and post-Omicron variants, modification of these glycan impact these variants uniquely. Removal of glycosylation sites in the S2, namely at N717 and N801, have profound impacts on Omicron variant pseudovirus infectivity, while only removal of N61 in S1 reduces infectivity of the D614G variant. The authors show that the presence of high mannose glycans ablates infectivity of Omicron variants, while having limited impact on pre-Omicron variants. Lastly the authors show that class 3 RBD mAbs are impacted by glycan modifications that favour the RBD up conformation of spike, while class 1 RBD mAbs are impacted by those modifications that favour the RBD down conformation.

What is lacking for me, is that the authors have not linked the findings from each set experiments to present a model or mechanism or reason for why, as their data suggests, there are functionally-dependent differences in glycosylation requirements for pre versus post-Omicron variants. The conclusion paragraphs that they authors have presented largely represents a summary of the results with implications that are very technical in their application.

**Authors’ reply:** We thank the reviewer for providing valuable feedback on our manuscript and have expanded the discussion section as suggested by reviewer. The modifications are highlighted in Part II below.

**Part II – Major Issues: Key Experiments Required for Acceptance**
Please use this section to detail the key new experiments or modifications of existing experiments that should be absolutely required to validate study conclusions.

Generally, there should be no more than 3 such required experiments or major modifications for a "Major Revision" recommendation. If more than 3 experiments are necessary to validate the study conclusions, then you are encouraged to recommend "Reject".

Reviewer #1: None noted.

Reviewer #2: I have suggested to strengthen Figure 4 as this is an important finding. See summary above.

**Authors’ reply:** We appreciate this comment and expanded Figure 4 to show the full growth curves. The results were obtained by titering the virus using the focus-forming assay, which in many ways is similar to a traditional plaque assay. The Omicron BA.1 does not plaque well, and many labs have turned to focus-forming assays to obtain more accurate titers. Cele *et.al.* previously demonstrated that infection by cell-free Omicron of unmodified Vero E6 cells was inefficient because of poor cytopathic effect (CPE) (Nature, 602, p654–656, 2022). This observation has been made by many other labs, including ours. Hence, a focus forming assay was developed to quantify the titer of Omicron BA.1.1 stock. We have added a sentence in the result sections with this explanation:

(lines 264-265)

Viruses were titered using a focus-forming assay due to poor cytopathic effects of the Omicron variant in Vero E6 cells.

Reviewer #3: Methods
Line 508: Given that the infectivity and relative infectivity measures are so critical to results, please provide more details as to how infectivity was measured and how relative infectivity was calculated. Is infectivity measured as the RLUs produced by a specific virus transfection? Were different aliquots or the same aliquot used for the p24 western blot and virus infectivity measures? Were multiple lots grown on different days compared?

VLP generation: Please include a section detailing how the SARS-CoV-2 VLPs were generated and quality checked.

**Authors’ Reply:** We thank the reviewer for this helpful feedback and have modified the methods section with more detailed descriptions of how the preparations of pseudoviruses/VLPs with different spikes were produced and characterized. We also added a supplementary figure (S3 Fig.) confirming comparable infectivity of the VLPs. The following sentences were included in the revision:

(lines 536-541)

Infectivity of each pseudovirus preparation was determined in 293T-ACE2/TMPRSS2 cells as previously described [58]. Briefly, 100 µL of pseudoviruses preparation was inoculated onto ACE2/TMPRSS2 plated at 3.0 x 10^4^ cells/well in a 96 well dish. The infectivity was determined by measuring luciferase activity 48 h later (luciferase assay system, Promega, Madison, WI) and normalized for the HIV-1 p24^CA^ in the inoculum. Results used at least two preparations of each pseudovirus and included at least two independent experiments.

(lines 555-567)

**SARS-Cov-2 VLP production and neutralization**

SARS-CoV-2 VLPs were produced by co-transfecting the following plasmids spike-pcDNA3.1(+) (8 ng), CoV2-M-IRES-E (1.65 µg), Luc-T20 (5 µg), and CoV2-N-WT-Hu1(3.35 µg) using Fugene 6 (Promega, Madison, WI) in a 10 cm dish containing 293T cells in 10% FBS/DMEM seeded the day before at 60-80% confluency. VLPs were harvested at 48 h, and supernatants were then filtered through a 0.45 µm low protein binding filters, aliquoted, and stored at -80°C until further use. Infectivity of each VLP preparation was determined in 293T-ACE2/TMPRSS2 cells. Briefly, equal volume of pseudoviruses dilutions (100 µL) were inoculated onto 96 well plates with ACE2/TMPRSS2 plated the day before at 3.0 x 10^4^ cells/well. The infectivity of the pseudovirus preparations were determined 48 h later (luciferase assay system, Promega, Madison, WI). The neutralization assays were preformed using 293T-ACE2/TMPRSS2 cells as previously described above. Results used at least two preparations of each pseudovirus and included at least two independent experiments.

Results
Line 436-444
This section comparing lentivirus to VLP is difficult to assess given the lack of details as to how the VLPs were generated. Regardless, results for a control antibody, that is able to neutralise the VLPs at greater than 30% maximum inhibition is required to demonstrate robustness of the assay.

**Authors’ Reply:** We thank the reviewer for the recommendation to add the control antibody. Data for neutralization with Bebtelovimab, which neutralizes 100% of all the pseudoviruses tested, was added to Figure 6. As described above, we also modified the methods section with a detailed description of how the VLPs were produced.

Discussion
The authors have included multiple points in their results section that could form the basis of a well-constructed discussion, which is currently lacking and which I believe will force the authors to tie together the many different points into some mechanistic and biologically relevant implications, rather than simply a set of technical recommendations.

**Authors’ Reply:** We appreciate this comment and expanded the discussion within the different sections to include mechanistic and biological implications. The following sections were expanded:

(lines 185-198)

The mechanisms underlying the differences in the glycans effects between pre-Omicron and Omicron variants are unclear. In high resolution structures of spike, the glycans are not fully resolved. We speculate that the large number of mutations, deletions, and insertions in the Omicron spike may alter spike folding and structure during biogenesis, which could interfere with the efficiency of glycosidases to trim and process glycans. These modifications could also affect spike stability and conformational dynamics. Comparison of the prefusion structures of the Wuhan-1 and BA.1 spikes suggests that the S2 mutations (N764K, D796Y, Q954H, N969K and L981F) do not lead to obvious structural changes [21]. In the pre-fusion conformation, glycans in residues N709, N717, N801, N1098 and N1134 form a glycan belt in the S2 region that shields the area between the S1 and the stalk (S2 Fig). The only Omicron mutation in this region is D796Y. In the post-fusion conformation, residues N709, N717 and N1098 remain nearly in the same position as in the pre-fusion conformation. However, residues D796 and N801 undergo a major shift in position in the post-fusion conformation. It is possible that the interactions between D796Y and glycan N801 affect the spike conformational changes.

(lines 236-247)

In summary, these results show that BA.1 and BA.2 spikes bearing only oligomannose glycans are poorly functional. Relative to D614G and Delta spikes, BA.1 and BA.2 spike function appears to be more sensitive glycosylation modifications. A prior study using lectin microarrays showed that Omicron variants have a high content of galactose-containing glycans and low content of high mannose glycans, suggesting a shift towards more complex or hybrid type of structures [24]. Changes in lectin binding could enable Omicron to interact with alternative receptors such as the DC-SIGN lectin [25]. SARS-CoV-2 has been reported to use DC-SIGN though it is not known if there are differences among the variants [26, 27]. Previously, the HIV antibody 2G12 was shown to bind to four high-mannose glycans at residues 709 and 801 in adjacent protomers in the Wuhan-1 spike [28], but 2G12 binding to the Omicron spike was weaker [29]. These reports align with our data suggesting that the glycan composition of Omicron spikes differs in important ways compared to spikes of earlier variants.

**Part III – Minor Issues: Editorial and Data Presentation Modifications**
Please use this section for editorial suggestions as well as relatively minor modifications of existing data that would enhance clarity.

Reviewer #1: It is unclear what the section on the VLPs is adding to this study.

**Authors’ Reply:** Lentiviral pseudoviruses capture spike when they bud from the plasma membrane, in contrast to live SARS-CoV-2 and the SARS-CoV-2 VLPs that bud from the ER-Golgi intermediate compartment. Different transits in the biosynthetic pathways could affect the glycosylation. We mentioned these points in the text (lines 456-460) Therefore, we used the VLPs as an extra control to confirm that the incomplete neutralization observed with some antibodies is not specific to the lentiviral pseudovirus platform.

Lines 330-333: “Both the apo and the S309 bound structures of the ancestral spike have an even proportion of closed and one-up RBD structures, but the one-up RBD conformation dominated the apo and S309-bound Omicron structures [48].” It is key to note here that the preference for one-up RBD in the Omicron spike is a result of the engineered 2P mutations, whereas without these mutations, the Omicron spike assumes a wider variety of conformational states (<https://pubmed.ncbi.nlm.nih.gov/35447081/>, <https://pubmed.ncbi.nlm.nih.gov/35452593/>). The authors should take this into account when discussing their data in the context of RBD up/down dynamics.

**Authors’ Reply:** We thank the reviewer for noting the important point regarding spikes with the 2P mutations. We added this point in the revision and included the relevant references:

(lines 353-358)

Both the apo and the S309 bound structures of the ancestral spike with the engineered two proline substitutions (2P) have an even proportion of closed and one-up RBD structures [45], but the one-up RBD conformation dominated the apo and S309-bound 2P engineered Omicron structures. Studies with the unmodified spikes show that it can assume a wide variety of conformational states [21, 29], and some Omicron substitutions seem to stabilize the closed conformation in a manner unique to Omicron [29].

-In line 13, “Short title: Effects of glycosylation in Omicron and D614 spikes differ.” Should be changed to “Short title: Effects of glycosylation in Omicron and D614G spikes differ.”

**Authors’ Reply:** We corrected the typo.

-Lines 79 and 81: “The protein sequence determines the position of the N-linked glycosylation, but the glycan occupancy and composition depend on the three-dimensional protein structure and the accessibility to glycan-processing enzymes.” Citations should be added here.

**Authors’ Reply:** Citations were added.

-When discussing the Li et al. paper and Huang et al papers (lines 89-91), it may be useful to add information on the expression system used in these studies.

**Authors’ Reply:** We agree and added a sentence stating that pseudoviruses were expressed in 293T cells.

-Line 132: What the authors mean here is “deletion of the glycosylation site at N801”.

**Authors’ Reply:** We thank the reviewer for noting that error and fixed the sentence.

-Line 187: The acknowledgement that “differences in local structure may also affect glycan site occupancy or glycan modifications” is appreciated, especially because a residue change in Omicron BA.1 occurs so close to the glycan site. A figure indicating this structural proximity will be a useful addition here.

**Authors’ Reply:** We thank the reviewer for the suggestion. We modified the text (lines 194-198) and added a new supplementary figure (S2 Fig) to expand this point. See also response to Reviewer #3 in Part II about addressing a mechanism (lines 185-198).

Reviewer #2: None

Reviewer #3: Title
I would recommend a modification to the title to read as “ Effects of N-glycan modifications on spike expression, virus infectivity and neutralization sensitivity in ancestral compared to Omicron SARS-CoV-2 variants”

**Author Reply:** We thank the reviewer for the suggestion and made the change.

Throughout the manuscript – “authentic virus”.
I would recommend that the authors refer to “authentic virus” as “live virus” or because in my opinion, there is nothing authentic about culturing SARS-CoV-2 in Vero E6 cells.

**Authors’ Reply:** We replaced “authentic virus” with the more commonly used term “live virus.” We had used the other term because some people prefer “authentic virus” to “live virus” because viruses are not living organisms.

Line 52
Suggest to alter the sentence to read as follows “Changes in the overall glycan composition also reduced the infectivity of Omicron compared to D614G in the live or pseudovirus format.”

**Authors’ Reply:** We appreciate the suggestion and made the change.

Line 67
The S2 subunit contains the transmembrane domain and contains other regions that do not span the transmembrane region. Therefore, please modify the sentence to read “….and a non-covalently associated viral fusion subunit (S2).”

**Authors’ Reply:** We now refer to S2 as the noncovalently-associated, viral fusion subunit (S2).

Line 119
Please clarify whether fold drop in infectivity was based on comparison to wild-type D614G (Fig 1C) or wild-type BA.1 (Figure 1D)?

**Author Reply:** We clarified the figure as suggested. It now the y-axes label reads “Infectivity relative to D614G” (Fig 1C) or “Infectivity relative to BA.1” (Fig 1D)

Line 204
The authors state that D614G and BA.1 had similar levels of infectivity, however in the western blots in both Figure 2 and Figure 3, the S1 bands for BA.1 appear to be much darker than for D614G. Can the authors comment on this relative to the infectivity read out?

**Author Reply:** We modified Figure 3B to show the infectivity of each variant relative to D614G. In the original Fig 3D, the blots had different exposure times making it difficult to compare the bands. We revised the figure using the same blots with similar exposure times, where it can be appreciated that the expression levels of S1 in D614G and BA.1 are comparable.

Line 227
The authors state that BA.1/BA/2 spikes depend on more “proper” glycosylation. Can the authors clarify what they mean by proper glycosylation or replace the term with “more processed glycans”?

**Authors’ Reply:** We replaced the term “proper” with “more processed glycans” as suggested.

Figures
Figure 1 legend
Please state what the dashed lines in panels C, D and E indicate?
Might make it easier for the reader if you indicate where the 10-fold and 100-fold difference in infectivity is for Fig 1, C, D and E.

**Authors’ Reply:** The figure and the legend have been modified as suggested to indicate e 10-fold and 100-fold difference in infectivity.

Figure 3A
Please include labels for the various glycoforms eg high mannose/oligomannose, hybrid or complex. Alternately, because for eg Man5-9GlcNAc2 is referred to in the text, these types of labels may also help orient the reader.

**Authors’ Reply:** Labels of the glycoforms have been added to the figure as suggested.

Figure 3 legend
There appears to be an error in the description for panel B and C. I assume panel B to represent results for multiple variants infectivity on the absence of glycosylation modifiers, however the current description appears to refer to panel C. In addition, the description for panel C only refers to mean and duplicates for each assay.

**Authors’ Reply:** We thank the reviewer for pointing this out and made the correction.
