# Supplementary material for: Effects of N-glycan modifications on spike expression, virus infectivity, and neutralization sensitivity in ancestral compared to Omicron SARS-CoV-2 variants
Source: PLoS Pathog. 2023 Nov 9;19(11):e1011788. doi: 10.1371/journal.ppat.1011788 (PMC10662749; doi:10.1371/journal.ppat.1011788)
Supplement: Attachment — Submitted filename: PPATHOGENS-D-23-00772 Bhiman Review 2.docx [file ppat.1011788.s007.docx]

**PPATHOGENS-D-23-00772 Revised**

**Effects of N-glycan modifications on spike expression, virus infectivity, and neutralization sensitivity in ancestral compared to Omicron SARS-CoV-2 variants**

**Part I - Summary**

As with the initial submission of this manuscript, the authors have generated a significant amount of data describing how glycosylation of the SARS-CoV-2 spike affects viral infectivity, spike processing and neutralization sensitivity. The inclusion of additional data/controls as well as details regarding the methodology has convinced me and addressed all my queries.

I like the description of the glycan belt and given that residues N801 and N796 are not resolved in the post-fusion spike structure, makes the role of these residues even more intriguing. Further, alterations in S2, including glycosylation, may affect overall conformation of S1, which in turn may affect for example receptor engagement. I find these data fascinating.

Lastly I thank the authors for addressing all comments and for being so generous in accepting my suggestions.

**Recommendation**

I would recommend that manuscript is accepted subject to very minor grammatical/spelling errors or pedantic preferences.

**Minor comments**

**Methods**

Line 565

“The neutralization assays were *preformed*…” should be “The neutralization assays were *performed*…”

**Figures**

Figure 1 panel E – I would change the y-axis title to “Infectivity relative to parental variant”

Figure 3 panel C - I would change the y-axis title to “Infectivity relative to untreated variant”

**Results**

Line 238

“...appears to be more sensitive glycosylation modifications.” Should be “...appears to be more sensitive to glycosylation modifications.”
